# Potential Cross-Links of Inflammation With Schizophreniform and Affective Symptoms: A Review and Outlook on Autoimmune Encephalitis and COVID-19

**DOI:** 10.3389/fpsyt.2021.729868

**Published:** 2021-09-16

**Authors:** Veronika Vasilevska, Paul C. Guest, Konstantin Schlaaff, Enise I. Incesoy, Harald Prüss, Johann Steiner

**Affiliations:** ^1^Department of Psychiatry, Otto-von-Guericke-University Magdeburg, Magdeburg, Germany; ^2^Laboratory of Translational Psychiatry, Otto-von-Guericke-University Magdeburg, Magdeburg, Germany; ^3^Laboratory of Neuroproteomics, Department of Biochemistry and Tissue Biology, University of Campinas, Campinas, Brazil; ^4^German Center for Neurodegenerative Diseases, Magdeburg, Germany; ^5^Institute of Cognitive Neurology and Dementia Research, Otto-von-Guericke-University, Magdeburg, Germany; ^6^German Center for Neurodegenerative Diseases, Berlin, Germany; ^7^Department of Neurology and Experimental Neurology, Charité—Universitätsmedizin Berlin, Berlin, Germany; ^8^Center for Behavioral Brain Sciences, Magdeburg, Germany; ^9^German Center for Mental Health, Center for Intervention and Research on Adaptive and Maladaptive Brain Circuits Underlying Mental Health, Magdeburg, Germany

**Keywords:** SARS-CoV-2, inflammation, schizophreniform disorder, affective disorder, neurological symptoms, autoimmune encephalitis

## Abstract

Based on current implications of the SARS-CoV-2 pandemic with regards to mental health, we show that biological links exist between inflammation and mental illness in addition to psychoreactive effects. We describe key principles of the biological interaction of the immune system and the mind, as well as the possible routes of viral entry into the brain. In addition, we provide a stepwise scheme for the diagnosis and therapy of autoimmune-encephalitis with schizophrenia-like symptomatology as a general guide for clinical practice and in the specialized scenario of infections, such as those caused by the SARS-CoV-2 virus.

## Introduction

The example of COVID-19 disease shows many aspects of how inflammation and mental illness can be linked. In the past year with the incessant progression of the COVID-19 pandemic, anxiety and depression occurred more frequently in the general population who had not been infected by the virus (1). In addition to existential and health concerns, the isolation induced by the necessary social restrictions and the countless lockdowns across the globe, were the most likely causative factors.

In a more direct way, some of those infected by the SARS-CoV-2 virus, the causative agent of COVID-19 disease, also developed reactive psychological complaints. These included the development of post-traumatic stress disorder in about 30% of cases who experienced a severe course of the disease and underwent intensive treatment (2). Mental and neurological disease symptoms resulting from direct disruption of brain function by the virus or secondary to the host immune response can also occur. SARS-CoV-2 has been detected in the brain by PCR and immunohistochemistry, but evidence to date suggests that it resides mainly in vascular and immune cells rather than directly infecting neuronal cells (3, 4). Olfactory loss, headache, alterations in consciousness and behavior, delirium, and agitation can be caused by the virus in this context. Secondary to the host immune response, cytokine-induced axonal degeneration, coagulopathies and blood-brain barrier disruption can occur with ischemic or hemorrhagic stroke, or autoantibodies can be produced potentially leading to autoimmune encephalitis or Guillain-Barré syndrome (4–6).

In this review, we present the links between immune system over-activation and psychiatric conditions such as schizophrenia and affective disorders. We also describe the new diagnostic entity in clinical neurology and psychiatry of autoimmune encephalitis with schizophreniform or schizoaffective symptoms which can occur due to the action of neuronal antibodies directed against synaptic receptors and cell surface proteins. Considering the devastating effects of the current pandemic on multiple aspects of physical and societal health, we place emphasis on the effects of the SARS-CoV-2 virus to disrupt immune system balance in the host with central nervous system (CNS) involvement and effects on mental health. Finally, we describe a systematic method including diagnostics and therapeutics of autoimmune-encephalitis with schizophrenia-like symptoms as guide for clinical practice and for cases of infections like COVID-19.

## Examples and General Principles of Biological Cross-Links of Inflammation With Schizophreniform and Affective Symptoms

### Cytokine-Induced Sickness Behavior

The so-called “cytokine-induced sickness behavior” has been known for decades. This can occur, for example, in response to an influenza infection, vaccination, an autoimmune or tumor-related disease. Kraepelin was the first to describe such changes during the influenza pandemic in 1890. He described 11 cases that developed psychiatric symptoms after infection, including depressive mood, paranoid, and hallucinatory symptoms, which suggested a link between the immune system and mental disorders (7). In this scenario, immune cells produce proinflammatory cytokines that act on the brain and cause depression-like mental and behavioral changes, such as depressed mood, emotional lability, poor concentration, loss of drive or motivation, social withdrawal, lack of appetite, sleep disturbances, and decreased personal hygiene (8). When activation of the peripheral immune system persists, as in chronic infections, autoimmune or tumor-related diseases, the release of pro-inflammatory cytokines such as interleukin (IL)-1, IL-6, interferon-gamma (IFNγ) and tumor necrosis factor alpha (TNF-α) in the CNS of predisposed individuals may cause persistence of disease with symptoms of depression. This can occur due to the fact that these cytokines decrease the availability of serotonin and its precursor tryptophan in the brain (9). Such changes, in addition to psychoreactive factors, could be partly responsible for the increased prevalence of depression in the above-mentioned somatic diseases.

### Cytokine Network Alterations in Schizophrenia and Affective Disorders

In addition to the above, changes in inflammatory cytokines have also been found in the blood of patients with Diagnostic and Statistical Manual of Mental Disorders (DSM)-defined psychiatric illnesses such as schizophrenia or affective disorders. For example, a meta-analysis of 68 studies found discretely elevated levels of the proinflammatory cytokines IL-6 and TNF-α in acutely ill patients with schizophrenia, bipolar disorder, and major depressive disorder (MDD) compared with non-psychiatrically ill control subjects (10). After treatment of the acute illness, IL-6 levels were found to be decreased significantly in both schizophrenia and MDD. In chronically ill patients, IL-6 levels were significantly increased in schizophrenia, euthymic bipolar disorder and MDD compared with controls. Overall, there were similarities in the pattern of cytokine changes in schizophrenia, bipolar disorder, and MDD during the acute and chronic phases of these illnesses, raising the possibility of common underlying immune dysfunction.

It should be mentioned that patients with acute onset of psychosis or depression often present poor hygiene and less self-care. For this reason, they tend to have more infections, especially those of an oral and dental nature, compared to a normal population (11). In addition, schizophrenia patients often develop metabolic syndrome (12). However, it is still unclear whether impaired cerebral glucose utilization leads to secondary disturbances in peripheral glucose metabolism and accompanying pro-inflammatory changes or whether initial immune disturbances drive the changes in glucose metabolism. IL-6 seems to induce insulin resistance and an increased production of CRP in the liver and TNF-α may also induce insulin resistance by suppressing the expression of the insulin-sensitive glucose transporter in peripheral tissues. Such potentially confounding factors have not been considered in most studies on the relationship of inflammation and schizophrenia (12). Furthermore, other studies have suggested the opposite effect. A Mendelian randomization study found that blockade of IL-6 cell signaling and low CRP levels can also be associated with a higher risk for schizophrenia (13). These contradictory effects might be explained by the ambivalent role of the IL-6 in the induction and resolution of inflammation. IL-6 can reduce the TGF-β-induced differentiation of Treg cells that inhibits autoimmunity. Thus, both downregulation or overproduction of IL-6 changes the balance between Th17 and Treg cells and can lead to development of autoimmune and chronic inflammatory diseases (14).

### Infectious Diseases and Autoimmune Disorders Increase the Risk for Manifestation of Schizophrenia and Affective Disorders

It is possible that the cytokine network changes presented above are an expression of a chronic mild inflammatory response due to an insufficient or misdirected immune response to pathogens, at least in a subgroup of patients (15, 16). Large epidemiological studies from Denmark found evidence that hospitalizations for infectious diseases or autoimmune disorders resulted in an increased risk for the manifestation of schizophrenia or affective disorder (17–19). It was reported that patients with schizophrenia and affective disorders show elevated antibody levels of measles (20) and Epstein-Barr (21) viruses compared to the control groups. In addition, serology studies with pregnant women showed that prenatal infection viruses such as rubella and influenza was correlated with the risk of developing schizophrenia (22). This study proposed that viruses change the development of the neuronal circuits in utero which can trigger an autoimmune reaction of the CNS. In line with this possibility, increased neutrophil granulocytes, monocytes, and proteins associated with the immune response to bacterial infections have been identified in the blood of acutely ill unmedicated schizophrenia patients (23). However, such evidence of infections or autoimmune responses in patients with mental illness should not be considered as monocausal in driving the disease, but rather in terms of a vulnerability-stress concept, such as an additional stressor or trigger in vulnerable individuals (e.g., genetic disposition or disruption of perinatal brain development).

### Neuropsychiatric Symptoms in Systemic Autoimmune Diseases

It has long been known that systemic autoimmune diseases affecting the CNS [e.g., systemic lupus erythematosus (SLE)], steroid-responsive encephalopathy in autoimmune thyroiditis (SREAT/Hashimoto's encephalopathy), or neuroinflammatory diseases such as multiple sclerosis or cerebral vasculitides, can lead to organic schizophreniform or affective symptomatology (24). In the case of SLE, both structural brain lesions caused by the autoimmune disease and diffuse brain dysfunction without structural lesions may play a role. Vasculopathies such as those associated with the presence of cardiolipin antibodies may cause cerebral (micro)infarcts visible on cerebral magnetic resonance imaging (MRI) analyses. However, in a considerable proportion of patients the imaging results were found to be unremarkable despite clear neuropsychiatric symptoms and diffuse slowing, whereas electroencephalography (EEG) recordings indicated generalized cerebral dysfunction (25). It is now known that in these cases, antibodies directed against neuronal cells and synapses play a role in the genesis of neuropsychiatric symptoms in addition to proinflammatory cytokines (see Cytokine-Induced Sickness Behavior). For example, some patients with SLE form autoantibodies due to a misdirected immune response against the GluN2 subunit (NR2) of the N-methyl-D-aspartate receptor (NMDAR) causing its overstimulation (26). This hyperactivation of the NMDAR-NR2 subunit leads to death of downstream neuronal cells and disruption of glutamatergic excitatory neurotransmission, which favors the occurrence of psychoses.

Some patients with autoimmune thyroiditis can also present various antibodies against neuronal cell surface proteins or synapse formations (27). These antibodies may enter the brain under multiple conditions such as immune-mediated (transient) alterations in blood-brain-barrier (BBB) integrity, caused by proinflammatory changes in monocytes and neutrophils, as well as *via* increased cytokine and C-reactive protein (CRP) blood levels (28, 29). With regards to development of antineuronal antibodies, it is assumed that molecular mimicry plays a role. This means that various pathogens partially adapt their proteins and carbohydrates to resemble those of the host in order to evade the immune system (30). However, this immune escape can lead to immunological cross-reactions with attacks on the body's own structures, in the scenario of an autoimmune reaction.

### Autoimmune Encephalitis With Mental and Behavioral Abnormalities

In recent years, a new diagnostic entity has been established in clinical neurology and psychiatry. Autoimmune encephalitis with schizophreniform or schizoaffective symptomatology due to antineuronal antibodies against synaptic receptors and surface proteins has been identified as a rare but potentially treatable cause of psychotic disorders (24, 31). The detection of specific IgG-class antineuronal antibodies is an important characteristic of these disorders and the exclusion of alternative causes may aid diagnostics and dictate more effective immunomodulatory therapeutic approaches (see section Stepwise Scheme for Diagnosis and Therapy of Autoimmune Encephalitis, below).

## Potential Role of Viruses in the Context of Autoimmune Encephalitis

### Viral Infections as a Predisposing Factor for NMDAR Encephalitis

In about 70% of cases with NMDAR encephalitis, neuropsychiatric symptoms have been observed to occur approximately within 2 weeks after a prodromal stage with flu-like symptoms such as headache, fever, loss of appetite, nausea or vomiting, or symptoms of an upper respiratory tract infection (32). For these reasons, viral diseases are potential triggers. It is known that NMDAR-encephalitides can occur after infection with viruses such as herpes simplex 1 or varicella zoster (33). The development of herpes encephalitis does not exclude the presence or the progression to autoimmune encephalitis, which should be treated with immunotherapy in the presence of positive antineuronal autoantibodies in the cerebrospinal fluid (CSF) after aciclovir treatment. Also, associations between herpes simplex virus 1 infections without encephalitis and the occurrence of NMDAR encephalitis have been reported (34). The production of antineuronal antibodies and encephalitis may occur as a secondary response to viral disease, although the detailed immunological cascade of autoimmunity after herpes simplex 1 encephalitis awaits experimental confirmation (35). Accordingly, production of antineuronal autoantibodies and encephalitis may occur as a secondary response to viral disease. If NMDAR autoantibodies are detected in the CSF, viral diagnostics should be performed as a precautionary measure to determine whether additional antiviral therapy is indicated. An association of influenza A seropositivity with the formation of NMDAR-NR1 antibodies in serum has also been described (36). In this case, cross-reactivity could arise because the M2 transmembrane protein of influenza A virus and NMDAR share a ligand, namely amantadine (37).

The Spanish flu pandemic in 1918–1920 resulted in an increased prevalence of mental illness. The most prevalent presentations were psychoses, catatonic symptoms, uncontrollable sleep attacks and temporary Parkinson-like symptoms, described as “encephalitis lethargica.” Molecular mimicry and cytokine-induced inflammation were discussed as possible triggering mechanisms (37). During and after the earlier Russian flu pandemic of 1889–1894, similar symptoms and an increased suicide risk were observed. In the majority of those affected, temporary insanity with depressive, psychotic, manic, or obsessive-compulsive symptoms was described shortly before the suicide attempt (38). An associated loss of the sense of smell has also been described (38), similar to some of the current effects of COVID-19 disease. In fact, there is now evidence that the Russian flu was not caused by an influenza virus but by the human coronavirus HCoV-OC43, which has since evolved into a relatively harmless cold virus. This finding is supported by data from a not-yet-published study by Lone Simonsen, an epidemiologist at Roskilde University in Denmark, who analyzed historical health data and worked with bioinformatics experts at the Technical University of Denmark to simulate viral mutation in reverse (https://nzzas.nzz.ch/wissen/coronavirus-er-hat-schon-vor-130-jahren-die-welt-gelaehmt-ld.1573590?reduced=true). Accordingly, new assumptions about the mutation rate of viruses, led the research group of Belgian virologist Marc Van Ranst at the University of Leuven to conclude that the virus had jumped from cattle to humans around the year 1890 (39). The researchers had compared gene sequences of HCoV-OC43 with bovine coronavirus (BCoV).

### Biological Mechanisms of Mental Disorders in the Context of COVID-19

In cases of psychiatric disorders associated with COVID-19 pathology, IL-6 and TH17 cells seem to play an important role (40, 41). TH17 cells are a special type of T helper cell that produces IL-17, activates neutrophil granulocytes, and is associated with the development of chronic inflammation and autoimmune diseases. IL-6 is one of the major inflammatory cytokines in infections with mucosal involvement. IL-6-dependent Th17 activation and differentiation is essential for neutrophil granulocyte migration (42). Experimental work showed that SARS-CoV-2-infected glial cells secrete increased levels of IL-6 and TNFα (5, 43). IL-6 can alter neuronal and glial activity and induce cell death, olfactory loss or induction of hyperphosphorylation of the tau protein in axons, triggering their degeneration. Several publications on SARS-CoV-2 report a strong association between high serum IL-6 levels (>80 pg/mL in serum) and severe disease progression or CNS involvement in COVID-19, even in the absence of respiratory symptoms (42). This strongly exceeds the reference values for serum IL-6 levels, which are typically 2.6–11.3 pg/mL (44). The newly developed guideline “Neurological manifestations in COVID-19—updated on 22.02.2021” of the German Society of Neurology (https://dgn.org/leitlinien/neurologische-manifestationen-bei-covid-19) mentions IL-2, IL-7, granulocyte colony-stimulating factor (GCSF) and TNF-α, in addition to IL-6 as potential biomarkers to estimate the risk of severe courses or encephalopathy. IL-6 elevation in serum seems to correlate with its increase in CSF. Reference values for this are laboratory-dependent but an elevation is said to occur at values above 5.9–7 pg/mL (42). The occurrence of affective and psychotic symptoms in the context of COVID-19 can be driven by elevated IL-6 levels which interfere with serotonergic and glutamatergic neurotransmission in the brain (45).

Another mechanism that may contribute to the development of COVID-19-associated neuropsychiatric symptoms was presented in a study by Yapici-Eser et al. (6). They described a possible mimicry between the NMDAR-NR1 subunit and viral nonstructural protein 8 (NSP8) and between the NMDAR-NR2 subunit and viral nonstructural protein 9 (NSP9), based on comparative analyses between the human genome and the RNA sequence of SARS-CoV-2. This mimicry may lead to the generation of IgG antibodies against the NMDAR subunits after SARS-CoV-2 infection. Recent case reports describing the development of NMDAR encephalitis in temporal association with SARS-CoV-2 positivity support this hypothesis (46–51). Activation of TH17 cells in COVID-19 sufferers may be a facilitating factor, as this has generally been associated with the occurrence of autoimmune diseases. Further evidence for cross-reactive autoantibodies in COVID-19 comes from studies using the binding of monoclonal patient antibodies to brain tissue (52) and high-throughput autoantibody discovery techniques (53).

Microvascular damage in the systemic inflammatory response to SARS-CoV-2 is thought to promote the occurrence of encephalopathy in severe COVID-19 disease (5). Inflammatory mediators produced by the alveolar epithelium, macrophages and leukocytes may contribute to endothelial inflammation, increased vascular permeability, edema, and increased synthesis and consumption of coagulation factors. As an example of the latter, elevated D-dimer levels can occur in patients with a severe COVID-19 course (54). Clots can trigger thromboembolic events of the cerebral arteries or sinus vein thrombosis. However, the increased vascular permeability also leads to disturbances of the microcirculation including BBB impairment (5). As mentioned above, BBB dysfunction may facilitate the entry of preformed or SARS-CoV-2-associated antineuronal autoantibodies from the periphery to the brain (28, 29). In autopsy reports of COVID-19 non-survivors, marked fibrinogen leakage from the small cerebral vessels and reactive perivascular macrophagocytosis have been noted despite the absence of viral particles in the brain tissue (55). Such secondary processes may become chronic and contribute to symptoms of long-COVID syndrome.

## Stepwise Scheme for Diagnosis and Therapy of Autoimmune Encephalitis

**Figure 1:**
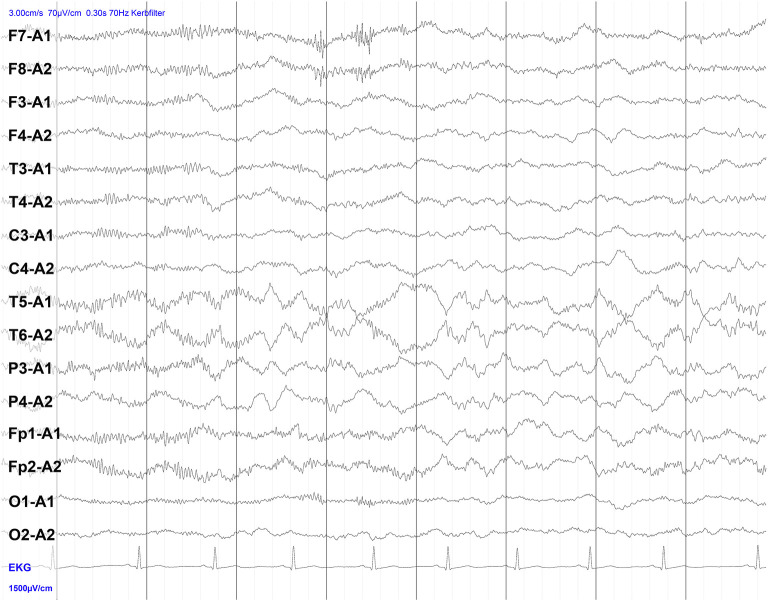
Electroencephalography (EEG) of a patient with NMDAR encephalitis. Intermittent bilateral delta activity with superimposed fast activity, a pattern that has been described as “extreme delta brush”-like (beta-delta complexes consisting of bilateral delta activity at 1–3 Hz and superimposed beta activity at 20–30 Hz). Note that the finding is less pronounced here than in classical descriptions of extreme delta brush.

For clinical practice, a step-by-step scheme has been developed which, guided by clinical warning signals, enables a rapid and reliable diagnosis and the initiation of immunotherapy (24, 31, 56, 57). In the case of subacutely evolving progressive psychiatric symptoms, the additional occurrence of focal neurological signs, disturbances of consciousness/orientation/memory, autonomic instability or epileptic seizures/EEG abnormalities, dictates the need for CSF analysis to investigate the potential presence of antineuronal autoantibodies, see Supplementary Data Sheet 1 (24, 31, 56, 57). For illustration, see Table 1; Figure 1 and our case report describing a patient with NMDAR encephalitis (IgG autoantibodies in CSF against the NR1a subunit of the NMDAR). This is the most common type of autoimmune encephalitis. Other specific antibodies with increased risk of psychosis are directed against the following synaptic and neuronal cell surface proteins: leucine-rich glioma inactivated 1 (LGI1; transmembrane protein associated with voltage-gated potassium channels), contactin-associated protein 2 (Caspr2; transmembrane protein associated with voltage-gated potassium channels), and the α-amino-3-hydroxy-5-methyl-4-isoxazole propionic acid receptor (AMPAR; ionotropic glutamate receptor), dipeptidyl peptidase-like protein-6 (DPPX; membrane protein which binds to voltage-gated potassium channels), γ-aminobutyric acid receptor (GABAR), metabotropic glutamate receptor 5 (mGluR5), and glycine receptor (GlyR; ionotropic, ligand-gated chloride ion channel) (24).

**Table 1:** Warning signs of autoimmune encephalitis in patients with psychotic symptoms and step-by-step diagnosis (31, 56, 58).

**Subacute onset (rapid progression within** ** <3 months) despite therapy plus:**	
• Disturbed consciousness	
• Movement disorder (dystonia or dyskinesia) or unsteadiness of stance and gait	
• Autonomic instability	
• Disorganized thinking/behavior	
• Catatonia/suspected malignant neuroleptic syndrome	
• Hyponatremia that cannot be explained by side effects of existing medication (SSRI, carbamazepine, etc.)	
• Epileptic seizures / faciobrachial dystonic seizures (the latter is common in LGI1 encephalitis)	
• Focal neurological deficits, including aphasia, dysarthria, or paresthesias	
• Newly developed headache or clinically relevant change in headache pattern	
• Prodromal flu-like symptoms	
• History of malignant tumor disease	
• Other autoimmune diseases (e.g., systemic lupus erythematosus, thyroiditis)	
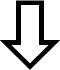 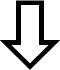	
**Further obligatory diagnostics**	
**cMRI** (MRI is unremarkable in ~50% of cases with autoimmune encephalitis)	• Hyper-intense signal in T2 or FLAIR sequences, mesiotemporally accentuated (limbic encephalitis), or multifocal in white and/or gray matter
**EEG**	• Epileptic or slow-wave activity, possibly temporally accentuated, extreme delta brush (beta-delta complexes consisting of bilateral delta activity at 1–3 Hz and superimposed beta activity at 20–30 Hz; this pattern is fairly typical for NMDAR encephalitis)
**Lumbar puncture/CSF analysis**Basic CSF diagnostics (cell count, albumin CSF/serum ratio, immunoglobulin index, oligoclonal bands)	• Lymphocytic pleocytosis (>5 cells/μl), specific oligoclonal bands, albumin CSF/serum ratio (blood-CSF-barrier disturbance). No evidence of infection but secondary autoimmune encephalitis may occur after viral encephalitis
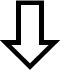 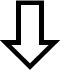	
**Measurement of antineuronal autoantibodies in cerebrospinal fluid and serum**	
**Basic antibody screening** minimally should include the most common IgG antibodies to the following antigens:	
• NMDA-R, CASPR2, LGI1, AMPA-R, GABAB-R, and GAD65 (determination in serum and CSF)	
• Hu, Ri, Yo, CV2/CRMP5, Ma2 [Ta], amphiphysin (determination in serum, CSF tests can be added if the serum is positive)	
In the **second step** (in cases of negative screening and reasonable suspicion), IgG antibodies can be detected against the following antigens:	
• GABAA-R, DPPX, mGluR5, Neurexin-3-alpha, IgLON5, and Glycin-R (determination in serum and CSF).	
**Immunofluorescence screening tests on rodent brain sections** (tissue-based assays) can also detect previously unknown antineuronal antibodies	
in specialized laboratories	

In an observational study of 501 NMDAR encephalitis patients (59), immunotherapy produced improvement in half of the cases within the first 4 weeks. Only 12% of all subjects relapsed within the first 24 months and only 4% had multiple relapses. Reactivation of the disease was usually milder than the first episode of encephalitis. A delay in therapy, as well as an emergence of patients requiring intensive care, was associated with a worse outcome and resulted in more relapses. Thus, although only a small group of psychiatric patients are affected by an autoimmune encephalitis, timely and correct diagnosis is of high therapeutic and prognostic relevance, as early and intensive immunotherapy often leads to a good prognosis despite severe disease.

Considering international expert recommendations, immunosuppression by corticosteroid therapy (1 g methyl-prednisolone/day for 5 days), intravenous human immunoglobulin administration (0.4 g/kg/day for 5 days) or immunoadsorption or plasmapheresis for rapid removal of pathogenic autoantibodies is first-line therapy in patients with definite autoimmune encephalitis (24). If therapy fails, treatment should be extended within a few days, preferably with rituximab (2 ×1,000 mg i.v. or s.c. at 2–4 week intervals). In refractory cases, combination treatment with cyclophosphamide (750 mg/m^2^ body surface area every 4 weeks), mycophenolate mofetil, or methotrexate may also be required to achieve a clinical response (24). Bortezomib may be a valuable option for escalation therapy (1–6 cycles of 1.3 mg/m^2^ body surface area for 21 days each cycle) for patients with NMDAR-encephalitis who require artificial ventilation in the intensive care unit and respond inadequately to standard immunosuppressive and B-cell depleting drugs (e.g., corticosteroids, IV immunoglobulins, plasma exchange, immunoadsorption, rituximab, and cyclophosphamide) (60). Bortezomib is a proteasome inhibitor that helps to eliminate plasma cells. In addition to clinical improvement, normalization of pathological cardiac MRI and EEG findings can be used to assess treatment success. Also, antineuronal serum and CSF antibody titres should decrease with successful therapy, which can be assessed after a few weeks of treatment (24).

Antipsychotics with low extrapyramidal side effects (quetiapine, clozapine) are particularly suitable for symptomatic pharmacotherapy of psychotic symptoms, as the risk of neuroleptic-induced dyskinesia or malignant neuroleptic syndrome is increased in patients with autoimmune encephalitis (24). Short-acting benzodiazepines can be used for anxiolysis and sedation and, at higher doses, to treat catatonic symptoms. Tumor screening using whole-body computed tomography (CT), MRI, positron emission tomography (PET), or transvaginal/testicular ultrasound should be performed when autoimmune encephalitis is detected, as antineuronal antibodies may be paraneoplastic (24, 56). Treatment of patients should be multidisciplinary and include psychiatrists and neurologists as well as immunologists and oncologists to afford the best chances of detecting and treating potential relapses in a timely manner.

## Summary, Conclusions, and Future Prospects for Precision Psychiatry

In this article, we reviewed information showing that inflammation and mental illness interact in multiple ways. For example, people affected by infectious, autoimmune, or tumor-related diseases may reactively develop psychiatric symptoms. To be distinguished from this are mental and neurological disease symptoms resulting from a direct disturbance of brain function by pathogens or by the immune defense system of the host. The latter is the cause of cytokine-induced sickness behavior which manifests with depression-like symptoms. An increased level of inflammatory cytokines, especially IL-6 and TNF-α, has also been found in the circulation of acutely ill patients with schizophrenia, bipolar disorder, and MDD.

It is possible that the cytokine network changes described in this report are an expression of mild inflammatory processes in the sense of an insufficient or misdirected immune response to pathogens, at least in patient subgroups. Indeed, epidemiologic studies suggest that prior hospitalization for infectious diseases and autoimmune disorders increases the risk for manifestation of schizophrenic and affective disorders. Systemic autoimmune diseases can lead to neuropsychiatric symptoms through various mechanisms. Using SLE as an example, we illustrated here that structural brain lesions due to vasculitic processes or cerebral (micro)infarcts, as well as diffuse brain dysfunction associated with elevated cytokines and antineuronal antibodies, may be involved. For instance, individuals with autoimmune thyroiditis produce antibodies against neuronal cell surface or synapses more frequently.

In line with this, we also described the emerging topic regarding diagnosis and immunotherapy of autoimmune encephalitis with psychotic symptoms induced by specific antineuronal antibodies. Our case vignette of a patient with NMDAR encephalitis vividly conveys an example of the clinical picture. Autoimmune encephalitis represents a rare but potentially treatable cause of psychotic and affective disorders and may be triggered by viral infections such as those caused by SARS-CoV-2. Although the mechanism is not precisely known, SARS-COV-2 infection of the brain appears to occur *via* retrograde transmission from the olfactory bulb or transport through a disrupted BBB (61), or it could occur indirectly in the sequelae of the cytokine storm effects (62). In all of these scenarios, the overproduction of inflammatory cytokines results in BBB disruption, allowing the further invasion of the brain by cytokines, monocytes and CD4+ lymphocytes bearing the virus. It should be noted that psychotic and affective symptoms are rare in cases of COVID or long COVID. The most common manifestations include fatigue and headache, although there is two-fold increased risk of developing a neuropsychiatric disorder after a COVID-19 diagnosis (63, 64). Autoimmune inflammation, such as Guillain-Barre syndrome or NMDAR encephalitis are described only in isolated cases and the causal association with SARS-CoV-2 is subject to conjecture at this time (5, 61).

Given the importance the potential role of viruses in the pathophysiology of neuropsychiatric illnesses such as autoimmune-encephalitis, it is critical that a correct diagnosis is made to allow the most appropriate treatment. Guided by clinical warning signals for autoimmune encephalitis and related syndromes, the step-by-step scheme highlighted in this review enables rapid and accurate diagnosis and initiation of therapy. The co-occurrence of psychiatric symptoms with focal neurological signs calls for CSF analysis to determine if neuronal autoantibodies are present. This is critical as timely diagnosis would allow early intervention which could improve disease outcome. Antipsychotics with low extrapyramidal side effects can be used for treatment of psychotic symptoms, and short-acting benzodiazepines can be used for anxiolysis, sedation, and treatment of catatonic symptoms. Tumor screening CT, MRI, PET, or ultrasound should be performed in confirmed cases since antineuronal antibodies may be paraneoplastic. Finally, treatment of patients should consist of multidisciplinary teams, comprised of psychiatrists, neurologists, immunologists, and oncologists to allow the best chances of managing the disease.

## Author Contributions

VV and JS drafted the first version of the manuscript. HP added the therapy section on autoimmune encephalitis and more information on viruses and autoantibodies. KS and EI helped with literature search and illustrations. PCG drafted the summary and abstract and edited the English language of the whole manuscript as a native speaker. All authors have read and agreed to the final version of this manuscript.

## Funding

This work has been partly supported by European Research Area Network (ERA-NET) NEURON Translational Biomarkers in Brain Disorders (Project NicAb, Grant No. 01EW2012).

## Conflict of Interest

The authors declare that the research was conducted in the absence of any commercial or financial relationships that could be construed as a potential conflict of interest.

## Publisher's Note

All claims expressed in this article are solely those of the authors and do not necessarily represent those of their affiliated organizations, or those of the publisher, the editors and the reviewers. Any product that may be evaluated in this article, or claim that may be made by its manufacturer, is not guaranteed or endorsed by the publisher.
