# Supplementary material for: Potential Cross-Links of Inflammation With Schizophreniform and Affective Symptoms: A Review and Outlook on Autoimmune Encephalitis and COVID-19
Source: Front Psychiatry. 2021 Sep 16;12:729868. doi: 10.3389/fpsyt.2021.729868 (PMC8507462; doi:10.3389/fpsyt.2021.729868)
Supplement: Supplementary file 1 [file Data_Sheet_1.doc]

**Case report**. Modified from supplementary information by .

A 27-year-old female patient presented as an emergency due to personality changes, memory and cognition deficits, speech disintegration, and disorientation. After a few days, the patient developed affect lability, anxiety, agitation, and paranoia. In the later course, catatonic symptoms, abnormal movements (orofacial dyskinesias, choreiform movement disorders, opisthotonus, myoclonus, and dystonic spasms of the extremities), autonomic instability with recurrent tachycardia, hyperthermia up to 39.3°C, and hypertension (160/100 mmHg) occurred. At this stage, MRI of the brain with T2-weighted and FLAIR images was unremarkable. EEG showed diffuse continuous activity in the theta range and bilateral delta activity with superimposed fast activity, a pattern described as "extreme delta brush"-like (**Figure 2**). A lumbar puncture was performed which revealed lymphocytic pleocytosis (21 cells/µL), an elevated IgG index (0.8), and positive oligoclonal bands. Virological and bacteriological diagnostics were unremarkable. With positive IgG autoantibodies against the NMDAR GluN1a subunit (NR1a) in serum (titer 1:1000) and CSF (titer 1:320), diagnosis of NMDAR encephalitis was made. Symptomatic therapy consisted of lorazepam, quetiapine, and valproate. Causal therapy was intravenous immunoglobulins (0.4 g/kg/day for 5 days) followed by plasma exchange and rituximab (375 mg/m^2^ weekly for 4 weeks) in combination with cyclophosphamide (750 mg/m^2^ given with the first dose of rituximab) since therapy was refractory. A left oophorectomy was performed for suspected ovarian teratoma, but histopathologic analysis was unremarkable. Symptoms improved after 6-12 weeks of treatment, correlating with a decrease in serum and CSF autoantibody titers. After rehabilitation, the patient exhibited only mild speech problems and deficits in learning and cognition. She has since fully recovered from her autoimmune disease, is employed and living with her family.

Reference:

Steiner, J., Walter, M., Glanz, W., Sarnyai, Z., Bernstein, H.G., Vielhaber, S., Kastner, A., Skalej, M., Jordan, W., Schiltz, K., Klingbeil, C., Wandinger, K.P., Bogerts, B., and Stoecker, W. (2013). Increased prevalence of diverse N-methyl-D-aspartate glutamate receptor antibodies in patients with an initial diagnosis of schizophrenia: specific relevance of IgG NR1a antibodies for distinction from N-methyl-D-aspartate glutamate receptor encephalitis. JAMA Psychiatry 70, 271-278.
